# TGF-β1 Induces Immune Escape by Enhancing PD-1 and CTLA-4 Expression on T Lymphocytes in Hepatocellular Carcinoma

**DOI:** 10.3389/fonc.2021.694145

**Published:** 2021-06-25

**Authors:** Shixiang Bao, Xiaopei Jiang, Shuai Jin, Peipei Tu, Jingtao Lu

**Affiliations:** School of Life Sciences, Anhui Medical University, Hefei, China

**Keywords:** hepatocellular carcinoma, transforming growth factor β1, T lymphocyte, programmed cell death 1, cytotoxic T lymphocyte-associated antigen-4

## Abstract

Primary liver cancer (PLC) is one of the most common types of cancer worldwide. Hepatocellular carcinoma (HCC) accounts for approximately 90% of PLC cases. The HCC microenvironment plays an important role in the occurrence and development of HCC. Immunotherapy for the HCC microenvironment has become an effective treatment strategy. T lymphocytes are an important part of the HCC microenvironment, and programmed cell death 1 (PD-1) and cytotoxic T lymphocyte-associated antigen 4 (CTLA-4) are the main immunosuppressive molecules of T lymphocytes. Transforming growth factor β1 (TGF-β1) can inhibit the immune function of T lymphocytes and promote the occurrence and development of tumors. However, few studies have explored whether TGF-β1 can upregulate the expression of PD-1 and CTLA-4 on T cells. In this study, we showed that TGF-β1 upregulated the expression of PD-1 and CTLA-4 on T lymphocytes and attenuated the cytotoxicity of T lymphocytes for HCC cells *in vitro* and *in vivo*. In addition, TGF-β1 increased the apoptosis of T lymphocytes induced by HCC cells. Finally, we found that the mechanism by which TGF-β1 upregulates the expression of PD-1 and CTLA-4 on T lymphocytes may be related to the calcineurin-nuclear factor of activated T cells 1 (CaN/NFATc1) pathway. This study will provide some experimental basis for liver cancer immunotherapy based on the tumor microenvironment.

## Introduction

Hepatocellular carcinoma (HCC) is the sixth most common type of human cancer and the second-leading cause of cancer-related death worldwide (1). Despite recent advances in the diagnosis and treatment of HCC, it remains a highly lethal disease (2). Transforming growth factor β1 (TGF-β1), a soluble cytokine produced and secreted mainly by inflammatory cells and malignant hepatocytes, is overexpressed in tumor tissue of most patients with HCC (3). Moreover, the overexpression of TGF-β1 correlated with the progression and prognosis of HCC (4).

T lymphocytes, belonging to one of the most important immune cell types in the HCC microenvironment, play an important role in the aggressiveness and progression of HCC (5). Programmed cell death 1 (PD-1) and cytotoxic T lymphocyte-associated antigen 4 (CTLA-4) are the main immunosuppressive signal molecules on the surface of T lymphocytes. PD-1, a specific receptor of programmed death-ligand 1 (PD-L1) and a type I transmembrane protein, is mainly expressed on activated T lymphocytes and negatively regulates their function (6). PD-L1 is the main ligand of PD-1, and its expression is enhanced in a variety of solid tumors. It mainly plays a role in weakening the function of local tumor-infiltrating T lymphocytes and reducing the production of killer cytokines. Following the binding of PD-1 to PD-L1, it provides stimulation signals for inhibiting the proliferation of activated T cells (7). Moreover, it inhibits the secretion of cytokines interleukin-1 (IL-1) and IL-2, promotes T cell apoptosis, mediates tumor immune escape, and promotes tumor growth (8–10). The PD-1/PD-L1 pathway can negatively regulate the immune response of activated T cells by inhibiting their proliferation and inducing apoptosis. The pathway also plays an important role in mediating the immune escape of HCC.

CTLA-4, another important costimulatory molecule, downregulates the function of T cells. It is mainly expressed on the surface of activated CD4+, CD8+ T lymphocytes, and B lymphocytes. It is involved in mediating T cell apoptosis and inhibiting T cell proliferation and cytokine expression, thereby exerting an immunosuppressive effect (11). Scheipers et al. (12) reported that CTLA-4 can mediate T cell apoptosis independently of the Fas pathway, reduce IL-2 secretion by T cells, and inhibit IL-2R expression, thereby inhibiting T cell proliferation. In addition, CTLA-4 also regulates the differentiation of T cells (13, 14). *In vitro* studies have suggested that CTLA-4 promotes the development of naive CD4+ T cells to T helper 1 cells (15). In contrast, blockage of CTLA-4 increases the responsiveness of T cells to common antigens, promotes T cell activation, improves the body’s anti-tumor response, and prevents the occurrence of tolerance (16).

Studies have shown that TGF-β1 promotes the occurrence and development of tumors by inhibiting the immune function of T lymphocytes (17, 18). However, whether TGF-β1 upregulates the expression of CTLA-4 on T cells remains unknown and only few studies have explored TGF-β1 upregulates the expression of PD-1 on T cells (19). In this study, we investigated the effect of TGF-β1 on the expression of PD-1 and CTLA-4 of T cells and on the development of HCC.

## Materials and Methods

### Materials

Recombinant human TGF-β1 was purchased from Proteintech Group (Chicago, IL, USA). Recombinant murine TGF-β1 was purchased from Novoprotein (Shanghai, China). PD-1, NFATc1, lamin B, and glyceraldehyde-3-phosphate dehydrogenase (GAPDH) antibodies and anti-mouse/rabbit secondary antibodies for Western blot were purchased from Proteintech Group. Phosphorylated-NFATc1 (p-NFATc1) antibody for Western blot was purchased from Bioss (Beijing, China). CTLA-4 antibody for Western blot and NFATc1 antibody for chromatin immunoprecipitation (ChIP) assay were purchased from Santa Cruz Biotechnology (Santa Cruz, CA, USA). Fluorescence-conjugated monoclonal antibodies (mAbs) to PD-1 and CTLA-4 were purchased from Bioss. Fluorescence-conjugated mAbs to CD3 and CD8 were purchased from BD Biosciences (San Jose, CA, USA). The TGF-β receptor (TGF-βR) inhibitor SB431542 and CaN inhibitor cyclosporin A (CsA) were purchased from TargetMol (Shanghai, China).

### *In Vitro* Cell Culture/Maintenance

The H9 human T lymphocyte cell line was purchased from the American Type Culture Collection (Manassas, VA, USA). The H22 murine HCC cell line (derived from balb/c mice) and SMMC-7721 and Bel-7404 human HCC cell lines were purchased from Shanghai Cell Bank, Chinese Academy of Science (Shanghai, China). All cell lines were cultured in RPMI 1640 Medium with 10% fetal bovine serum, 100 U/mL penicillin, and 100 μg/mL streptomycin in a humidified atmosphere containing 5% CO_2_ at 37°C.

### Animals

Male Chinese Kunming (KM) mice (body weight: 18–22 g) were provided by the Experimental Animal Center of Anhui Medical University (Hefei, China) (No. SCXK-Wan-2011-002). KM mice first originated from Swiss mice are widely produced and are widely used in pharmacological studies in China (20). KM mice are a type of immunocompetent mice with normal immune function. Animals were maintained in a pathogen-free environment (23 ± 2°C, 55 ± 5% humidity) on a 12-h light/dark cycle throughout the experimental period. All animal studies were approved by the Institutional Animal Care and Use Committee of Anhui Medical University.

### Cell Treatment

H9 cells were activated for 48 h with 25 μL/mL CD3/CD28 T cell Activator (STEMCELL, Germany) and 150 ng/mL IL-2 (Sangon Biotech, Shanghai, China) in complete RPMI 1640 medium. Activated H9 cells were seeded in six-well plates at a density of 2×10^6^ cells/mL. Subsequently, TGF-β1 (5, 10, and 20 ng/mL) was added, and the cells were incubated for 48 h. The same medium without TGF-β1 was added to the control group. Following treatment with TGF-β1, the H9 cells were harvested and used for the following experiments. In the blockage experiments, TGF-βR inhibitor (SB431542, 10 μM) or CaN inhibitor (CsA, 1 μM) was added to the culture.

### Flow Cytometry

Cells were harvested and washed twice with phosphate-buffered saline (PBS). Next, the expression of PD-1 and CTLA-4 on H9 cells was identified using phycoerythrin anti-human PD-1-specific mAb and fluorescein isothiocyanate anti-human CTLA-4-specific mAb according to the instructions provided by the manufacturer. After gentle mixing, the samples were incubated in the dark for 30 min at 4°C and analyzed using flow cytometry. The fluorescently labeled cells were analyzed using the FlowJo analysis software (Tree Star, Ashland, OR, USA). The appropriate isotype control antibodies were used, and the results were reported as the mean fluorescence intensity.

### Western Blot Analysis

H9 cells were harvested and lysed in lysis buffer. Protein concentrations were measured using the bicinchoninic acid protein assay kit (Vazyme, Nanjing, China), followed by heating under reducing conditions. A total of 30 µg of protein was loaded on sodium dodecyl sulfate-polyacrylamide gel electrophoresis gels, and subsequently transferred onto polyvinylidene difluoride membranes. After blocking, the membranes were incubated with the following antibodies: GAPDH (1:10,000), lamin B (1:1,000), PD-1 (1:1,000), CTLA-4 (1:200), NFATc1 (1:1,000), and p-NFATc1 (1:10,000).

### Real-Time Quantitative Polymerase Chain Reaction Assay

Total RNA was extracted from H9 cells under the indicated conditions using the RNA Pure Plus Kit (TIANGEN, Beijing, China). Extracted RNA (100 ng) was reverse-transcribed using the FastKing RT Kit (TIANGEN). The generated cDNA was subjected to real-time qPCR assay to determine the mRNA expression levels of target genes. All target genes were normalized to the expression of GAPDH mRNA. The following primers were used in this experiment. Forward: 5’- GCCACCTTCACCTGCAGCTTGT -3’ Reverse: 5’- AAACCGGCCT TCTGGTTTGGGC -3’ (PD-1); Forward: 5’- CTACCTGGGCATAGGCAACG -3’ Reverse: 5’- CCCCGAACTAACTGCTGCAA -3’ (CTLA-4); Forward: 5’- GGAGCGAGATCCCTCCAAAAT -3’ Reverse: 5’- GGCTGTTGTCATACTTCTCATGG -3’ (GAPDH).

### Cell Counting Kit-8 Assay

HCC cells seeded in 24-well plates (1×10^4^ cells/well) and cultured in a 5% (volume/volume) CO_2_ humidified incubator at 37°C. The following day, TGF-β1-treated H9 cells and control group H9 cells were added to the 24-well plates (2×10^5^ cells/well) to be co-cultured with HCC cells for 24 h. Subsequently, the medium was discarded, and the wells were washed thrice with PBS to remove H9 cells. Next, 400 μL of medium and 40 μL of CCK-8 agent were added to each well, and the cells were incubated in the dark for 2 h. Finally, the absorbance of each well was determined at 450 nm.

### Lactate Dehydrogenase Cytotoxicity Assay

HCC cells were seeded in 24-well plates (1×10^4^ cells/well) and cultured in a 5% (volume/volume) CO_2_ humidified incubator at 37°C. The following day, TGF-β1-treated H9 cells and control group H9 cells were added to the 24-well plates (2×10^5^ cells/well) to be co-cultured with HCC cells for 6 h. Next, the LDH Cytotoxicity Assay Kit (Beyotime, Beijing, China) was used to test the cytotoxicity of H9 cells for HCC cells.

### Colony Formation Assay

HCC cells were seeded in six-well plates (1×10^3^ cells/well) in triplicates. The following day, TGF-β1-treated H9 cells and control H9 cells were added to the six-well plates (1×10^5^ cells/well) to be co-cultured with HCC cells. The medium and H9 cells were replaced every 3 days. After incubation for 12 days, the plates were washed twice with PBS, fixed using methanol for 10 min, and stained with 0.1% crystal violet solution for 10 min for further analysis.

### Analysis of H9 Cells Apoptosis

H9 cells (1×10^6^ cells/well) were collected from the co-culture system and washed twice with cold PBS. Subsequently, H9 cells were centrifuged and resuspended in 500 μL of PBS. Annexin V and propidium iodide staining buffer was added according to the protocol of the cell apoptosis detection kit. After gentle mixing, the cells were incubated in the dark for 10 min and analyzed using flow cytometry.

### ChIP Assay

H9 cells were crosslinked with 1% formaldehyde for 10 min, followed by quenching with glycine. Sonication was performed to obtain genomic DNA fragments of approximately 200–400 bp. Sonicated DNA was immunoprecipitated with 2.0 µg anti-AR antibody (Santa Cruz Biotechnology) at 4°C overnight. Immunoglobulin G was used in the reaction as a negative control. Target sequences within the human PD-1 promoter and CTLA-4 promoter were detected by PCR with the following specific primes. Forward: 5’- CCTCACATCTCTGAGACCCG -3’ Reverse: 5’- CCGAAGCGAGGCTAGAAACC -3’ (PD-1 promoter); Forward: 5’- GAGGACCCTTGTACTCCAGGAA -3’ Reverse: 5’- CGAAAAGACAACCTCAAGCACTC -3’ (CTLA-4 promoter).

### *In Vivo* Injection of H22 Cells

Exponentially growing H22 cells were subcutaneously injected into the right armpit of KM mice (2.5×10^6^ cells/mouse). Each group included eight animals. At day 3, mice were injected with TGF-β1 (70 ng/mL or 280 ng/mL) or normal saline *via* the tail vein once daily at a dosage of 5 mL/kg. At day 14, mice peripheral blood samples were collected from the retro-orbital sinus and the mice were humanely sacrificed. Then, the tumors were removed and weighed. The expression of PD-1 and CTLA-4 on CD8+ T cells in peripheral blood were analyzed by flow cytometry.

### Statistical Analysis

The data were expressed as the mean ± standard deviation from at least three independent experiments. Statistical analysis was performed using the statistics software GraphPad Prism 10 (GraphPad Software, Inc., La Jolla, CA). Comparisons between two and multiple groups were performed using Student’s *t*-test and one-way analysis of variance, respectively. *P<*0.05 denoted statistically significant differences.

## Results

### TGF-β1 Enhanced the Expression of PD-1 and CTLA-4 on Activated T Cells

**Figure 1:**
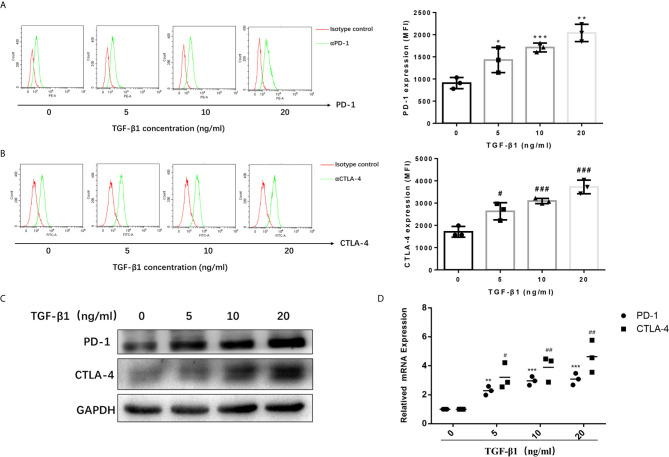
TGF-β1 enhances the expression of PD-1 and CTLA-4 on activated T cells. T cells were activated and treated with TGF-β1 (0, 5,10, 20 ng/mL) for 48 h. **(A)** The expression of PD-1 on T cells was analyzed by flow cytometry. **(B)** The expression of CTLA-4 on T cells was analyzed by flow cytometry. **(C)** The protein expression levels of PD-1 and CTLA-4 in T cells were analyzed by western blotting. **(D)** The mRNA levels of PD-1 and CTLA-4 in T cells were analyzed by qPCR. n = 3, *P<0.05, **P<0.01, ***P < 0.001 *vs.* control group; *^#^P* < 0.05, *^##^P* < 0.01, *^###^P* < 0.001 *vs.* control group.

To access the effects of TGF-β1 on PD-1 and CTLA-4 expression, we used H9 cells, a normal human T lymphocyte cell line, and activated them into effector T cells with CD3/CD28 T cell Activator and IL-2 (**Figures S1**, **S2**). Then, we treated activated H9 cells with TGF-β1 across a range of concentration (0, 5, 10, 20ng/mL) for 48h. The results of flow cytometry (**Figure 1A**) and Western blot (**Figure 1B**) showed that TGF-β1 significantly enhanced the expression of PD-1 and CTLA-4 on T cells. The qPCR results also showed that TGF-β1 significantly increased the mRNA levels of PD-1 and CTLA-4 (**Figure 1C**). Furthermore, we found that TGF-β1 upregulated the expression of PD-1 and CTLA-4 in a concentration-dependent manner (**Figure 1**).

### TGF-β1 Attenuated the Cytotoxicity of T Cells for HCC Cells

**Figure 2:**
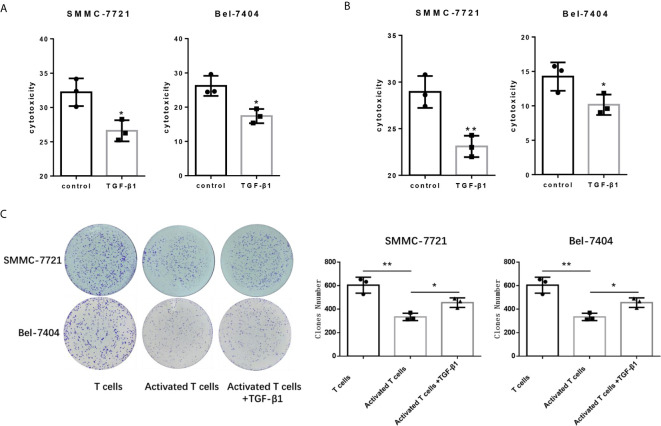
TGF-β1 attenuates the cytotoxicity of T cells for HCC cells. **(A)** Activated T cells stimulated or not stimulated with TGF-β1 were co-cultured with SMMC-7721 cells or Bel-7404 cells for 24 h. CCK-8 assay was used to determine the cytotoxicity of T cells for SMMC-7721 and Bel-7404 cells. **(B)** LDH cytotoxicity assay was used to determine the cytotoxicity of T cells for SMMC-7721 and Bel-7404 cells. **(C)** Colony formation assay was used to determine the cytotoxicity of T cells for SMMC-7721 and Bel-7404 cells. n = 3, *P < 0.05, **P < 0.01 *vs.* control group.

Since PD-1 and CTLA-4 are the main immunosuppressive molecules on the surface of T cells, which negatively regulate T cells functions. Next, we tested the cytotocicity of T cells for HCC cells after TGF-β1enhanced the expression of PD-1 and CTLA-4. We compared the capacity of T cells with or without TGF-β1 treated to kill HCC cells by co-cultivating T cells with HCC cells. T cells co-cultured with tumor cells are pre-activated, and then treated with TGF-β1 or PBS for 48 hours before co-cultivation, and then the treated T cells were co-cultured with HCC cells. We used SMMC-7721 and Bel-7404 HCC cell lines which express PD-L1 (**Figure S3**). The CCK-8 and LDH cytotoxicity assays showed that TGF-β1 attenuated the cytotoxicity of T cells for SMMC-7721 cells and Bel-7404 cells (**Figures 2A, B** **)**. The results of the colony formation assay also indicated that TGF-β1-treated T cells exhibited weaker cytotoxicity for SMMC-7721 cells and Bel-7404 cells compared with untreated T cells (**Figure 2C**). In addition, microscopic observation of the clone shape revealed that SMMC-7721 cells co-cultured with unactivated T cells formed approximately round, complete clones; SMMC-7721 cells co-cultured with activated T cells formed irregular clones. However, the clones of SMMC-7721 cells co-cultured with activated T cells stimulated by TGF-β1 have a tendency to restore round, complete shape (**Figure S4**).

### Apoptosis of TGF-β1-Treated T Cells Was Increased After Co-Culture With HCC Cells

**Figure 3:**
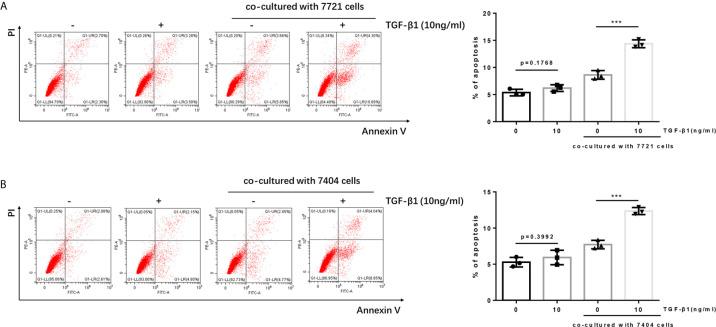
Apoptosis of TGF-β1-treated H9 cells is increased after co-cultured with HCC cells. **(A)** TGF-β1-treated/untreated activated T cells were co-cultured with SMMC-7721 cells or cultured separately for 24 h; subsequently, the apoptosis rate of T cells was detected by flow cytometry. **(B)** TGF-β1-treated/untreated activated T cells were co-cultured with Bel-7404 cells or cultured separately for 24 h; subsequently, the apoptosis rate of T cells was detected by flow cytometry. n = 3, ***P < 0.001 *vs.* control group.

It is reported that the PD-1/PD-L1 pathway can mediate the apoptosis of T cells (21, 22). When PD-1 on T cells recognizes and binds to PD-L1 on tumor cells, it will cause T cells to initiate the apoptosis mechanism, thereby allowing tumor cells to escape. Since we have determined TGF-β1 enhanced the expression of PD-1 on T cells. Next, we tested whether TGF-β1 treated T cells had higher apoptosis level after co-cultured with HCC cells. The results of flow cytometry showed that the apoptosis of T cells treated with TGF-β1 and subsequently co-cultured with HCC cells was significantly increased. However, the apoptosis of T cells treated with TGF-β1 but not co-cultured with HCC cells was only slightly increased and was not a statistically significant change. This finding demonstrated that the T cell apoptosis in the co-culture system was caused by co-cultivation with tumor cells rather than treatment with TGF-β1 (**Figure 3**).

### TGF-β1 Upregulated the Expression of PD-1 and CTLA-4 on CD8+ T Cells and Promoted HCC Growth *In Vivo*

**Figure 4:**
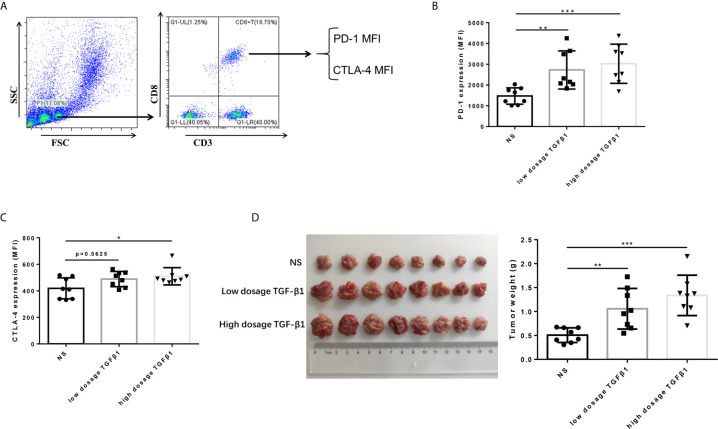
TGF-β1 upregulates the expression of PD-1 and CTLA-4 on CD8+ T cells and promotes HCC growth *in vivo*. **(A)** Flow cytometry was used to isolate CD3+ CD8+ T cells from peripheral blood mononuclear cells and detect the fluorescence intensity of PE-conjugated PD-1 protein and FITC-conjugated CTLA-4 protein on the cell surface. **(B)** Flow cytometry was used to detect the expression of PD-1 on CD8+ T cells in the peripheral blood of mice with HCC. **(C)** Flow cytometry was used to detect the expression of CTLA-4 on CD8+ T cells in the peripheral blood of mice with HCC. **(D)** Subcutaneous tumors extracted from Kunming mice 14 days after implantation, and the weight of these tumors. n = 3, *P < 0.05, **P < 0.01, ***P < 0.001 *vs.* control group.

To determine the effect of TGF-β1 on the expression of PD-1 and CTLA-4 on T cells and its effect on the development of HCC *in vivo.* We subcutaneously transplanted H22 cells, a mouse-derived HCC cell line, to KM mice, and injected mouse-derived TGF-β1 (70ng/mL, 280ng/mL) or normal saline *via* tail vein three days after inoculation, once a day with the dosage of 5mL/kg. The mice were sacrificed 14 days after transplantation. The results showed that the expression of PD-1 and CTLA-4 on CD8+ T cells in peripheral blood of TGF-β1 mice was significantly increased (**Figures 4B, C** **)**, and the tumors grew faster compared with those observed in mice injected with normal saline (**Figure 4D**).

### TGF-β1 Promoted the Expression of PD-1 and CTLA-4 on T Cells *via* TGF-βR/CaN/NFATc1 Signals

**Figure 5:**
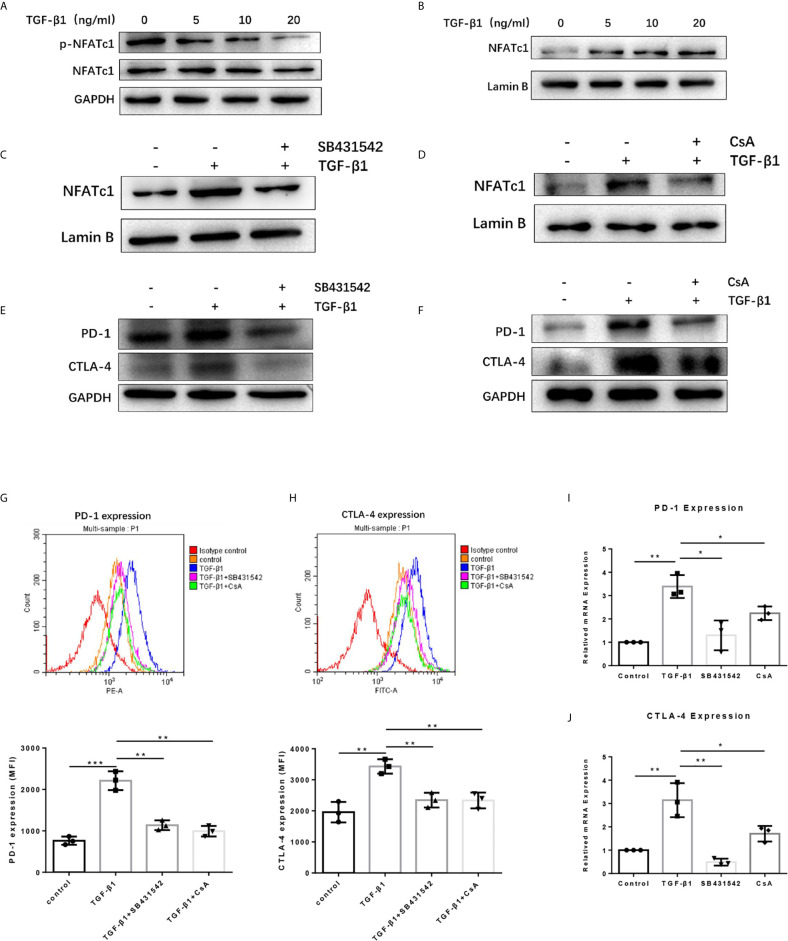
TGF-β1 promotes the expression of PD-1 and CTLA-4 on T cells *via* TGFβR/CaN/NFATc1 signals. **(A)** Western blot was used to detect the expression of total NFATc1 protein and phosphorylated NFATc1 protein in T cells. **(B–D)** Western blot was used to detect the expression of NFATc1 protein in the nucleus of T cells. **(E, F)** Western blot was used to detect the protein expression of PD-1 and CTLA-4 in T cells. **(G)** Flow cytometry was used to detect the expression of PD-1 on T cells. **(H)** Flow cytometry was used to detect the expression of CTLA-4 on T cells. **(I)** qPCR was used to detect the mRNA level of PD-1 in T cells. **(J)** qPCR was used to detect the mRNA level of CTLA-4 in T cells. n = 3, *P < 0.05, **P < 0.01, ***P < 0.001 *vs.* control group.

To dissect the mechanisms by which TGF-β1 increased PD-1 and CTLA-4 expression on T cells, we used the Western blot assay to determine some proteins related to the TGF-β/Smads pathway and PD-1 and CTLA-4 expression. The results revealed that TGF-β1 reduced the phosphorylation level of NFATc1 protein in T cells (**Figure 5A**). NFATc1 protein is generally located in the cytoplasm in a highly phosphorylated state. Its role is to be dephosphorylated by calcineurin (CaN) and then translocate to the nucleus to regulate gene transcription. Further analysis showed that TGF-β1 increased the level of NFATc1 protein in the nucleus of T cells (**Figure 5B**). Next, we used SB431542, a TGF-β receptor (TGF-βR) inhibitor and Cyclosporin A (CsA), a CaN inhibitor to treat H9 cells. Western blot results showed that both SB431542 and CsA inhibited the nuclear entry of NFATc1 increased by TGF-β1 (**Figures 5C, D** **)** and the expression of PD-1 and CTLA-4 enhanced by TGF-β1 (**Figures 5E, F** **)** in T cells. The flow cytometry results (**Figures 5G, H** **)** and qPCR results (**Figures 5I, J**) also showed that SB431542 and CsA inhibited PD-1 and CTLA-4 expression induced by TGF-β1 in T cells. Thus, these results suggest that TGF-β1 may promote the expression of PD-1 and CTLA-4 on T cells *via* TGF-βR/CaN/NFATc1 signals.

### NFATc1 Directly Targeted the Promoter Regions of PD-1 and CTLA-4 Genes

**Figure 6:**
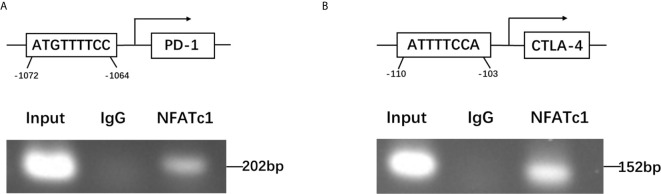
NFATc1 directly targets the promoter regions of the PD-1 and CTLA-4 genes. **(A)** ChIP assay was used to detect the binding of NFATc1 to the promoter region of the PD-1 gene. **(B)** ChIP assay was used to detect the binding of NFATc1 to the promoter region of the CTLA-4 gene.

To further dissect the mechanism by which NFATc1 regulate PD-1 and CTLA-4 expression at a molecular level, bioinformatic analysis using Ensembl and PROMO 3.0 was performed to search for potential NFATc1 response elements in PD-1 and CTLA-4 promoter regions. The potential NFATc1 response elements were found within 1.5 kb upstream of the transcriptional start site. Investigation by ChIP assay showed that NFATc1 could bind to the NFATc1 response elements located on the promoters of the PD-1 and CTLA-4 host genes (**Figures 6A, B** **)**.

### Blockage of the TGF-βR or CaN/NFATc1 Pathway Recovered the Cytotoxicity of H9 Cells

**Figure 7:**
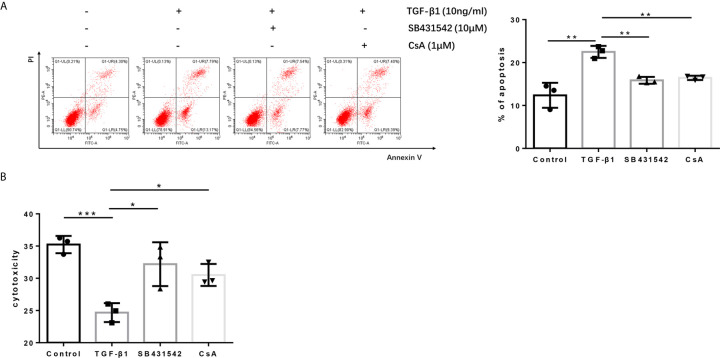
Blockage of the TGF-β receptor or CaN/NFATc1 pathway recovers the cytotoxicity of H9 cells. **(A)** Flow cytometry was used to detect the apoptosis of T cells after co-culture with Bel-7404 cells for 24 h. **(B)** CCK-8 reagent was used to detect the cytotoxicity of T cells for Bel-7404 cells after co-culture with Bel-7404 cells for 24 h. n = 3, *P < 0.05, **P < 0.01, ***P < 0.001 *vs.* control group.

Since blockage of the TGF-βR or CaN/NFATc1 pathway decreased the expression of PD-1 and CTLA-4 enhanced by TGF-β1, we next investigated whether SB431542, a TGF-βR inhibitor, and CsA, a CaN inhibitor, can inhibit the apoptosis upregulated by TGF-β1 and restored T cell cytotoxicity weakened by TGF-β1. Further analysis showed that both SB431542 and CsA inhibited T cell apoptosis upregulated by TGF-β1 and restored T cell cytotoxicity weakened by TGF-β1 (**Figures 7A, B** **)**.

## Discussion

The HCC microenvironment, which is composed of HCC cells, lymphocytes, hepatic stellate cells, epithelial cells, cytokines, and other interstitial components, plays an important role in the occurrence and development of HCC (23). HCC immunotherapy for the HCC microenvironment has become a new treatment strategy. T lymphocytes are an important part of the tumor microenvironment. CD8+ T cells in the circulatory system infiltrate the tumor tissue and are activated by tumor antigens to become effector CD8+ T cells with the function of killing tumor cells. Activated CD8+ T cells are also termed cytotoxic T lymphocytes. These lymphocytes recognize tumor antigens and subsequently release granzymes and activate Fas/FasL pathways to promote tumor cell apoptosis, or exert direct/indirect anti-tumor effects through the release of cytokines (24). PD-1 and CTLA-4 are the main immunosuppressive signal molecules on the surface of T lymphocytes, which can inhibit their function. TGF-β1, an inhibitory cytokine in the tumor microenvironment, also inhibits the function of T lymphocytes.

H9 cell, a normal human T lymphocyte line, can be activated into effector T cells that kill tumor cells using a CD3/CD28 antibody. In this study, we used H9 cells to conduct *in vitro* experiments. We found that TGF-β1 promoted the expression of PD-1 and CTLA-4 in T cells *in vitro* and *in vivo*. PD-1 can inhibit the proliferation of activated T cells, induce T cell apoptosis, and regulate T cell generation (8–10). CTLA-4 plays an immunosuppressive effect by mediating T cell apoptosis and inhibiting T cell proliferation and cytokine expression (12). Next, we used TGF-β1 to stimulate T cells and co-cultured these cells with HCC cells to test whether the killing effect of H9 cells on HCC cells was weakened. The present findings revealed that TGF-β1 attenuated the killing effect of T cells on SMMC-7721 and Bel-7404 cells. Moreover, the flow cytometry results showed that the apoptosis of TGF-β1-treated T cells was increased after co-culture with HCC cells.

We also investigated the mechanism by which TGF-β1 enhances the expression of PD-1 and CTLA-4 on T cells. Firstly, we tested whether the TGF-β/Smads pathway was activated and participated in the expression of PD-1 and CTLA-4. Interestingly, the results showed that the levels of p-Smad2 and p-Smad3 did not increase and showed a tendency to decrease. We hypothesized that the activation of other TGF-β pathways inhibited the TGF-β/Smads pathway. Subsequently, we tested whether NFATc1 is involved in the expression of PD-1 and CTLA-4 induced by TGF-β1. NFATc1 is an important member of the nuclear factor of the activated T cell (NFAT) family (25). NFAT is a critical nuclear transcription factor, which regulates the transcription of PD-1 and CTLA-4 genes (26, 27). The results showed that the level of p-NFATc1 in T cells was significantly decreased after stimulation with TGF-β1. NFATc1 is usually located in the cytoplasm of immune cells in a highly phosphorylated state. Following stimulation of the cells, NFATc1 can respond to Ca2^+^-calmodulin signals by dephosphorylation and translocate to the nucleus (28, 29). Subsequently, the results showed that the level of NFATc1 in the nucleus of T cells was significantly increased after stimulation with TGF-β1. ChIP assay results revealed that NFATc1 can directly target the promoter regions of the PD-1 and CTLA-4 genes and promote their transcription. The dephosphorylation of the NFAT family is specifically mediated by CaN. We also found that TGF-β1 did not increase the expression of CaN. However, the CaN inhibitor CsA blocked the increased transportation of NFATc1 to the nucleus and enhanced the expression of PD-1 and CTLA-4 induced by TGF-β1 in T cells. Therefore, we hypothesized that TGF-β1 increases the activity of CaN instead of increasing the expression of CaN.

**Figure 8:**
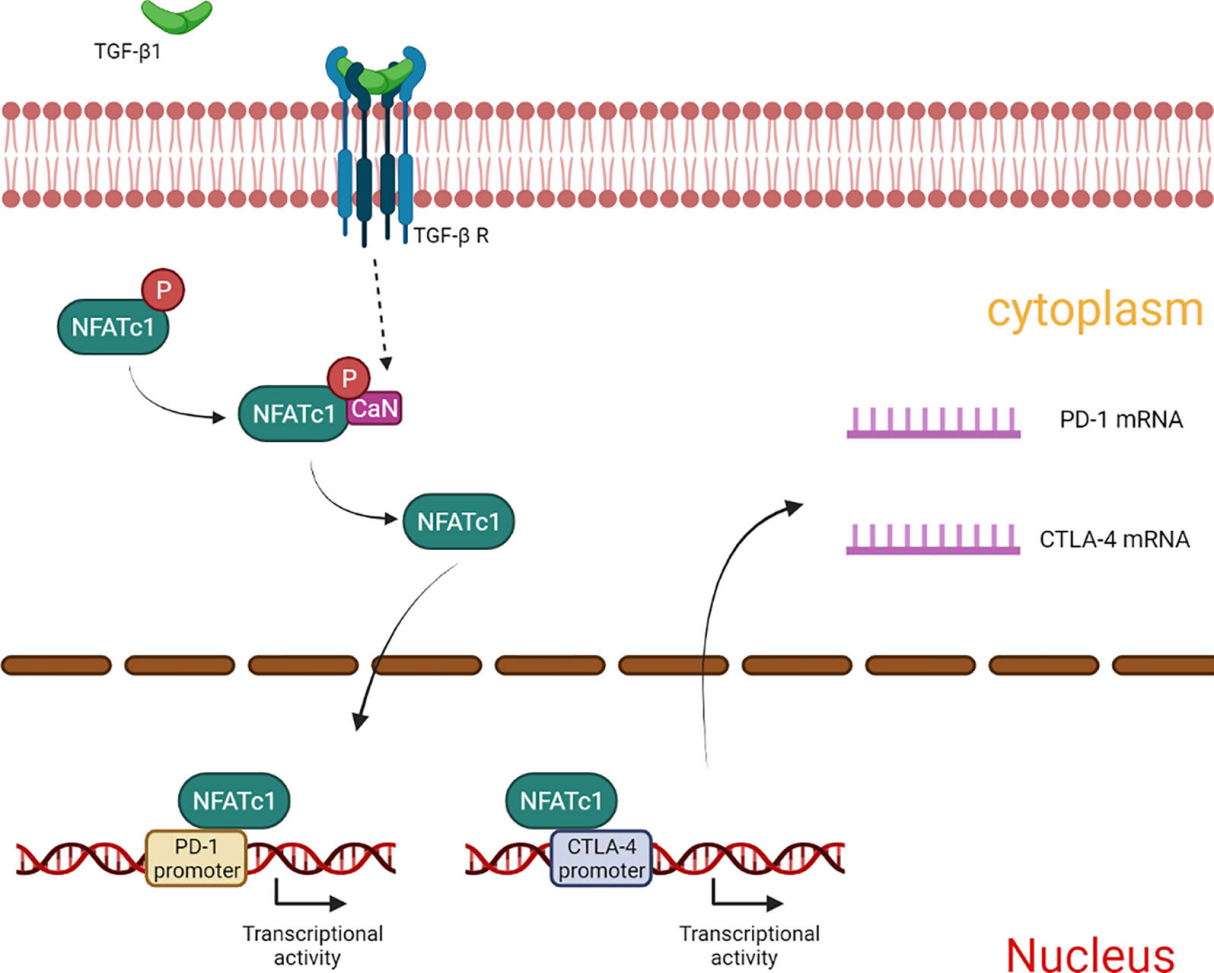
Summary of the mechanism by which TGF-β1 up-regulates the expression of PD-1 and CTLA-4 in T cells.

In summary, our results showed that TGF-β1 enhances the expression of PD-1 and CTLA-4 on T cells, and attenuates T cell cytotoxicity for HCC cells. The mechanism involved in this process may be related to CaN/NFATc1 signaling (**Figure 8**). In this study, we supplemented the new mechanism by which TGF-β1 up-regulates PD-1 expression on T cells and found for the first time that TGF-β1 also up-regulates the expression of CTLA-4 on T cells. This study provides some new experimental basis for HCC immunotherapy targeting TGF-β1.

## Data Availability Statement

The raw data supporting the conclusions of this article will be made available by the authors, without undue reservation.

## Ethics Statement

The animal study was reviewed and approved by ethics committee of Anhui Medical University.

## Author Contributions

All authors contributed to the article and approved the submitted version.

## Funding

This work was supported by the National Natural Science Foundation of China (81773105) and Natural Science Foundation of Anhui Province (1708085MH181).

## Conflict of Interest

The authors declare that the research was conducted in the absence of any commercial or financial relationships that could be construed as a potential conflict of interest.
