# Supplementary material for: TGF-β1 Induces Immune Escape by Enhancing PD-1 and CTLA-4 Expression on T Lymphocytes in Hepatocellular Carcinoma
Source: Front Oncol. 2021 Jun 25;11:694145. doi: 10.3389/fonc.2021.694145 (PMC8270637; doi:10.3389/fonc.2021.694145)
Supplement: Supplementary file 1 [file Image_1.pdf]

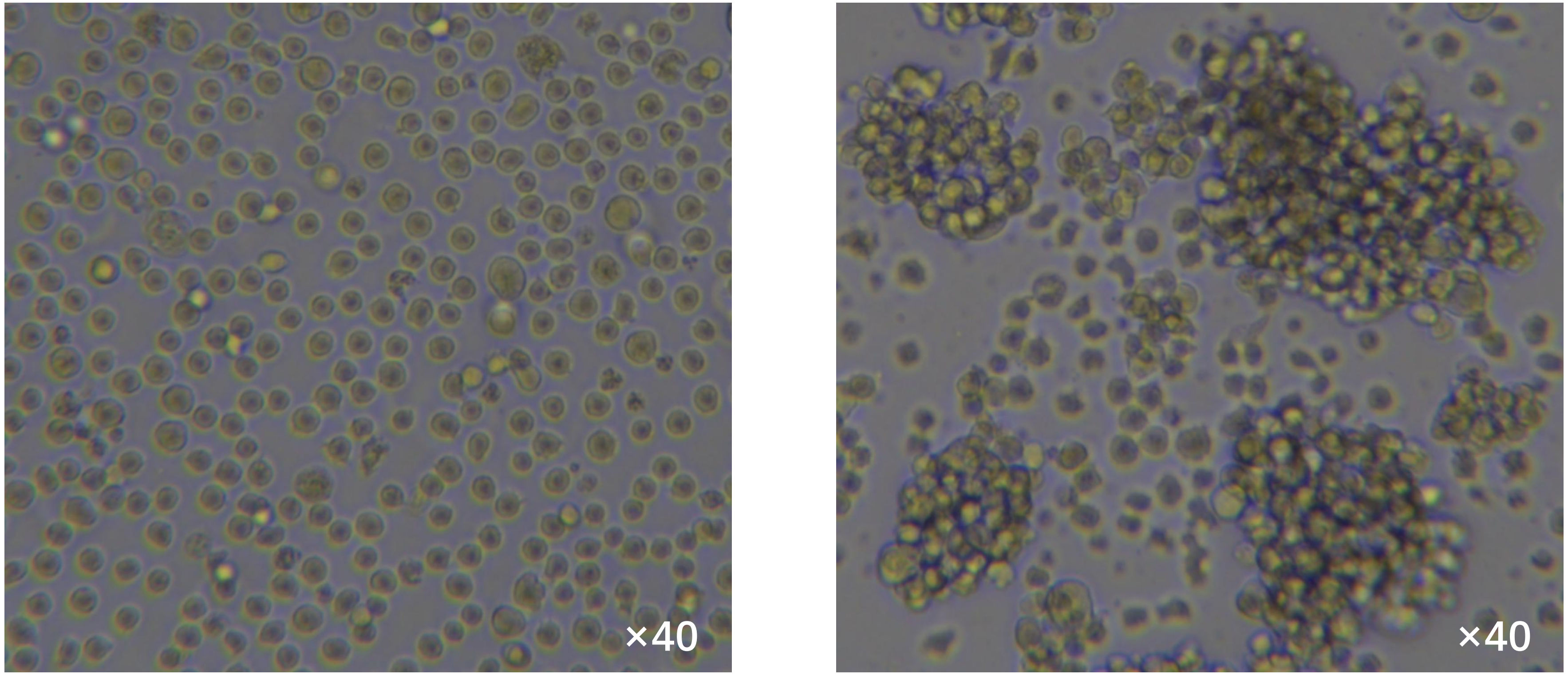

unactivated T cells

activated T cells

Fig.S1 Treating T cells with αCD3/CD28. After treating H9 cells with αCD3/CD28, H9 cells showed agglomeration, which is one of the phenomena that T cells are activated.

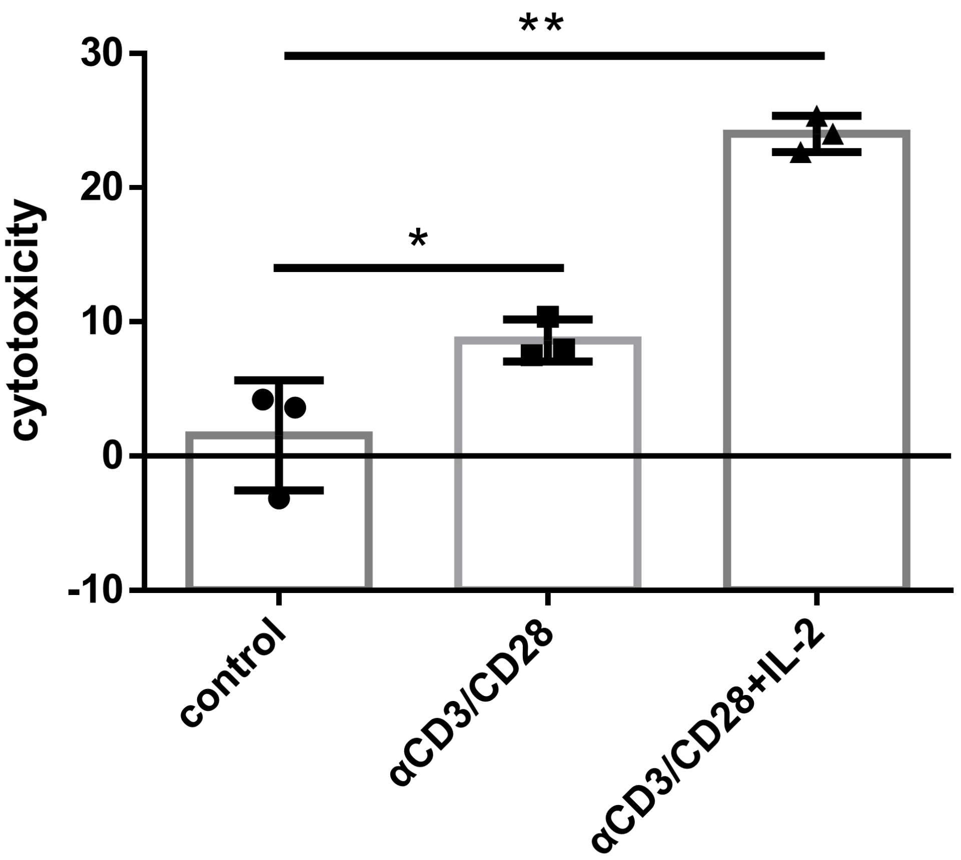

Fig.S2 CCK-8 assay detects the cytotoxicity of T cells for 7721 cells. We tested the cytotoxicity of H9 cells and found that the cytotoxity of H9 cells for SMMC-7721 cells was significantly enhanced after treated with αCD3/CD28. Furthermore, H9 cells treated with αCD3/CD28 and IL-2 together had stronger cytotoxicity. Therefore, we consider that H9 cells can be activated as effector T cells with tumor-killing functions.

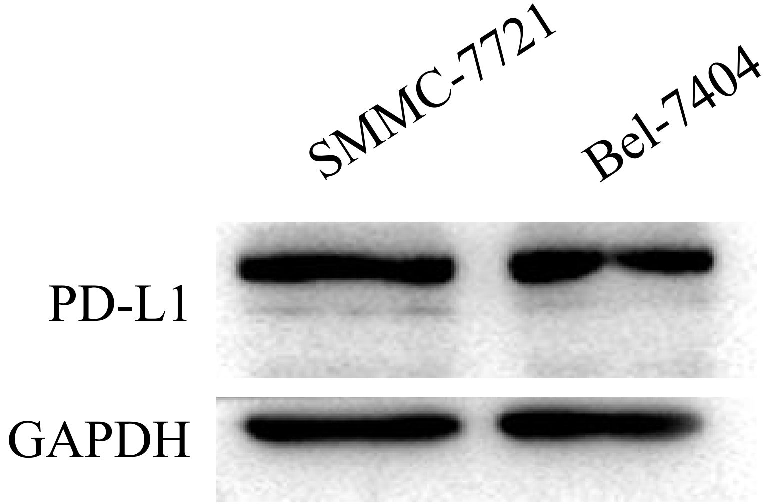

Fig.S3 Western blot was used to detect the expression of PD-L1 protein of SMMC-7721 and Bel-7404 cells.

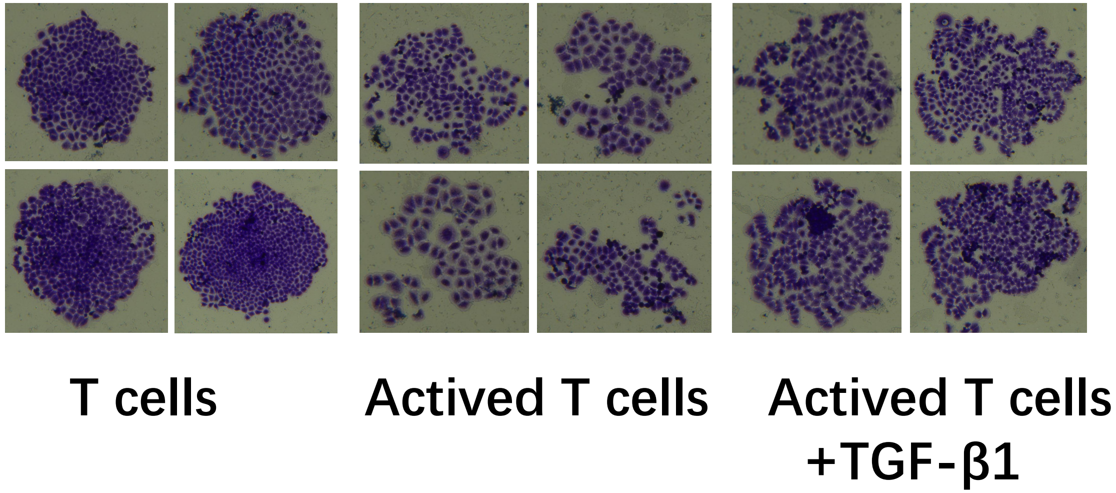

Fig.S4. The shape of clones of SMMC-7721 cells co-cultured with T cells.
